# Three Potential Sources of Microfungi in a Treated Municipal Water Supply System in Sub-Tropical Australia

**DOI:** 10.3390/ijerph8030713

**Published:** 2011-03-03

**Authors:** Noel B. Sammon, Keith M. Harrower, Larelle D. Fabbro, Rob H. Reed

**Affiliations:** 1 Centre for Plant and Water Science, CQUniversity Australia, Bruce Highway, Rockhampton, Queensland, 4702, Australia; E-Mail: r.reed@cqu.edu.au (R.H.R.); 2 Centre for Environmental Management, CQUniversity Australia, Bruce Highway, Rockhampton, Queensland, 4702, Australia; E-Mail: l.fabbro@cqu.edu.au

**Keywords:** airborne spores, artificial coupons, biofilm, Calcofluor White, inner pipe surfaces, sediments, frog, *Litoria caerulea*

## Abstract

Some microfungi are known to be opportunistic human pathogens, and there is a body of scientific opinion that one of their routes of infection may be water aerosols. Others have been implicated as causative agents of odours and off-tastes in drinking water. This study was undertaken to investigate three potential sources of microfungi in a treated, oligotrophic municipal water supply system in sub-tropical Australia. Formation of the microfungal component of developing biofilm on hard surfaces in water storage reservoirs was also assessed. Inside and outside air samples were collected from two reservoirs using two types of Burkard air samplers. Biofilm and soft sediment samples were collected from the inner surfaces of asbestos cement water pipes and from pipe dead ends respectively. These were analysed for microfungal growth and sporulation using Calcofluor White stain and epifluorescent microscopy. Artificial coupons of glass, PVC and concrete were immersed in two reservoirs to assess microfungal biofilm formation. This was analysed periodically using Calcofluor White stain and epifluorescent microscopy, cultures of coupon swabs and scanning electron microscopy. Fungal spores were recovered from all air samples. The number of colonies and the genera were similar for both inside and outside air. Microfungal filaments and sporulating structures were recovered from most of the pipe inner surface biofilm and dead end sediment samples, but were sparser in the biofilm than in the sediment samples. No recognisable, vegetative filamentous fungi were found in the slowly developing biofilm on coupons. This study indicates that airborne spores are an important potential source of microfungi found in water storage reservoirs. It has also demonstrated conclusively that filamentous microfungi grow and sporulate on water pipe inner surfaces and in soft sediments within the water distribution system.

## 1. Introduction

A number of microfungi are known to be opportunistic human pathogens [1] while others have been implicated in the production of odours and off-tastes in water [2]. There is a growing body of opinion that water aerosols containing microfungal spores, particularly those of *Aspergillus* spp., which are generated by hospital showers and taps, may be one of the sources of nosocomial mycotic infections. However, the role of water as a route for human infection has not yet been established [3–8]. Those most at risk of systemic fungal infection are patients whose immune systems have been compromised by diseases such as HIV AIDS or diabetes mellitus, and those who are treated with immunosuppressive drug therapies. In Australia most, if not all, hospital water supplies are sourced from treated municipal water systems and generally do not receive any secondary treatment. Consequently, if nosocomial mycotic infections are eventually linked to inhalation of spore-bearing water aerosols, then the microfungal populations of municipal water supply systems and their potential sources will generate considerable interest from water authorities and the scientific community. At present, the Australian Drinking Water Guidelines [9] do not contain any regulations covering microfungi.

Only a limited number of studies into the incidence of microfungi in municipal water supply systems have been published world-wide [10–22] and only one in Australia [23], but these have established that microfungi are ubiquitous in water supply systems, regardless of the sources of raw water or the treatment processes applied to that water. These studies have concentrated on quantification and characterisation of microfungal contaminants, and little attention has been directed to the sources of those contaminants. Some of the possible sources of initial and continuing contamination may include airborne spores introduced into water storage reservoirs from the external environment, proliferation of microfungi in biofilm on distribution pipe inner surfaces and in pipe dead end sediments, and passage through the treatment plant processes.

The first published study of the microfungal component of biofilm in drinking water systems was conducted by Nagy *et al.* [24] who investigated biofilm development in a water aqueduct system in Los Angeles USA. They found that filamentous microfungi are a component of such biofilm. Doggett [25] reported that fungal spores are common constituents of biofilm in water distribution systems. Kelley *et al.* [17] detected microfungi in biofilm which formed on artificial coupons. However, none of those studies provided conclusive evidence of the presence of vegetative and sporulating microfungi in the biofilm.

There are no standard methods which conclusively detect filamentous fungal growth in biofilm on the inner surfaces of water supply infrastructures and in soft bottom sediments which accumulate within the system. Furthermore, the accurate quantification of filamentous microfungi in those environments is impossible for the reasons outlined and discussed by Paterson and Lima [26]. FISH and Calcofluor White staining techniques as alternative means of detecting *in situ* filamentous fungi in water biofilm were described by Gonçalves *et al.* [27].

Any method of recovery which involves disturbance of the biofilm, such as swabbing or scraping, will undoubtedly result in fragmentation of some existing microfungal colonies, and the resulting mycelial fragments are potential propagules. Consequently, if quantification of microfungal colony-forming units (CFU) in such samples is assessed by counting the number of colonies which grow on culture plates prepared from aqueous suspensions of those samples, then interpretation of the results could be prone to considerable error. Furthermore, colonies which grow on the plates may have originated from allochthonous spores which have become trapped in the biofilm or in the water boundary layer at the solid surface/water interface. It is therefore not possible to determine, by these methods, whether or not any microfungi are actually growing and sporulating in the biofilm. In this study, and for those reasons, we used methods involving microscopic visualisation of biofilm components stained with Calcofluor White fluorochrome for direct detection of microfungal vegetative growth and sporulation as well as the conventional methods mentioned.

We have previously demonstrated that microfungi are recoverable from all parts of the Rockhampton water supply system [23]. The objective of this study was to investigate whether or not the source(s) of those microfungi may be airborne spores introduced into water reservoirs from the external environment, or from sporulation of filamentous microfungi growing in biofilm on hard surfaces within the system and in soft sediments in pipe dead ends. Formation of the microfungal component of developing biofilm on artificial coupons immersed in the oligotrophic water of storage reservoirs was also assessed. We have already established that, in areas where it is endemic, the large Australian green tree frog *Litoria caerulea* introduces yeasts and filamentous microfungi as well as the bacterium *Escherichia coli* into water storage reservoirs [28,29]. Whether or not some microfungal spores pass through the treatment plant, or survive the water treatment processes in a damaged state but are subsequently capable of recovering within the distribution system, is being separately investigated. The water supply system, the subject of this study, is the sole source of reticulated drinking water in the sub-tropical city of Rockhampton, Central Queensland, Australia which has a population of *ca.* 70,000.

## 2. Materials and Methods

### 2.1. Standard Materials and Methods

Unless otherwise stated, the following materials and methods were used in all experiments discussed in this paper:

*Culture plates*—Malt extract agar with chloramphenicol (MEAC) (bacteriological agar 10 g, malt extract 10 g, glucose 10 g, peptone 0.5 g, chloramphenicol 50 mg, reverse osmosis water 500 mL) in standard plastic Petri dishes.

*Micro-filtration*—Millipore^™^ filtration units using Millipore^™^ 0.45 μm pore size, 47 mm diameter, black S-Pak cellulose filter membranes.

*Calcofluor White stain*—Calcofluor White M2R 1 g in 1 L of sterile reverse osmosis water (SROW).

*DAPI stain*—4′,6-Diamidino-2-phenylindole dihydrochloride 5 μg in 1 mL SROW.

*Acid fuchsin stain*—Acid fuchsin 0.1 g in 100 mL of lactic acid.

*Enumeration and identification*—Culture plates were incubated at 25 °C in the dark, and colonies were counted and identified to genus level over 7 days by the micromorphology of their reproductive structures and spores and by reference to Barnett and Hunter [30]; Carmichael *et al.* [31]; Ellis [32,33]; Gilman [34] and Kendrick and Carmichael [35].

*Microscopy*—All light and epifluorescent microscopy was done with a Leica DMLB microscope. For all epifluorescent microscopy, filter cube D (UV + violet excitation filter BP 355–425 nm wavelength and suppression filter LP 470 wavelength) was used. Scanning electron microscopy was done with a Jeol Model JSM-6360LA scanning electron microscope (SEM). Stereomicroscopy was done with a Wild M3S stereomicroscope.

*Photography*—Photographs were taken with a Nikon Coolpix 995 digital camera. Photomicrographs were taken with an Olympus ColorView IIIu digital camera mounted on the Leica microscope. Composite photographic figures were assembled using Adobe Photoshop and Illustrator software.

### 2.2. Specific Materials and Methods

#### 2.2.1. The Source Water

Raw water for the water supply treatment plant is obtained from a riverine impoundment with a storage capacity of *ca.* 80,000 megalitres of surface run-off from a catchment area of *ca.* 139,000 km^2^. Summer rainfall in the catchment ensures strong annual river flows with major flood events occurring every few years. The catchment includes a variety of soil types and supports a wide range of natural vegetation and various grazing and agricultural activities. The littoral areas of the impoundment and the associated inter-connected lagoon systems are heavily endowed with aquatic grasses and other plants. The nature of the catchment and storage areas are thus conducive to a build-up of the microfungal population through rainwater run-off and airborne deposition of fungal propagules from surrounding areas. The copiotrophic raw water is naturally highly turbid during summer river flows with the very fine suspended colloids settling slowly through the winter months when the river flow diminishes or ceases.

#### 2.2.2. The Treatment Process

One water treatment plant services the whole distribution system. The raw water is treated by coagulation/flocculation with polyaluminium chlorhydrate and a polymer followed by sedimentation, sand filtration and sanitisation with chlorine to leave a residual free chlorine level of *ca.* 0.60 ppm before leaving the treatment plant. The raw water is also treated with activated carbon when cyanobacterial blooms occur in the river. Activated carbon is used to remove taste and odour compounds (e.g., geosmin) which can also be produced by fungi in the system. The treated water is pumped through trunk mains to 12 above-ground, roofed storage reservoirs and distributed through a system of distribution mains to consumers. The storage reservoirs are pulse-filled as demand dictates.

#### 2.2.3. The Distribution System

The water supply system dates back to *ca.* 1890. Consequently, the distribution system comprises a composite pipe network of different materials, including cast iron, cement-lined cast iron, asbestos cement and PVC which were introduced as the system expanded, materials improved, and repairs and replacements were carried out.

#### 2.2.4. Airborne Fungal Spores

Internal and external air was sampled at two roofed, concrete water storage reservoirs, designated R3 and R4, using two types of Burkard volumetric air samplers (Burkard Manufacturing Co., Ltd., Rickmansworth, Hertfordshire, United Kingdom). One type, the Burkard Personal Volumetric Air Sampler, is designed for trapping airborne particulate matter on glass microscope slides. The other type, the Burkard Personal Volumetric Portable Air Sampler, is designed for use with agar-based culture plates [36]. The manufacturer’s rated capacity of each air sampler is 10 L of air per minute. Each microscope slide used was finely coated on one side with white petroleum jelly (Vaseline^®^) by spreading a solution of one part petroleum jelly in 99 parts petroleum spirit (v/v) over the surface. The petroleum spirit quickly evaporated leaving a very thin coating of petroleum jelly in which the airborne spores could be trapped.

Air samples from inside the reservoirs were collected at a distance of 0.5 m above the water level, and outside air samples were collected on the roofs of both reservoirs. Thirty litres of air were passed through each of the samplers and five replicates were collected at each sampling event. Immediately after removal from the samplers, the microscope slides were placed in capped slide holders, the culture plates were lidded and placed in plastic bags to prevent contamination, and both were placed in pre-cooled insulated containers for transport to the laboratory.

At the laboratory, a drop of acid fuchsin stain was placed on each microscope slide, a cover slip was applied, and fungal spores were counted immediately under a light microscope. The culture plates were incubated and resulting microfungal colonies were counted under a stereomicroscope. A needle-point sample of each filamentous fungal colony was placed in a drop of acid fuchsin stain on a microscope slide, a cover slip was applied, and the sample was examined and identified to genus level under a light microscope. This accords with the identification level used in our earlier published work on the same water system [23]. Future studies should identify microfungi to species level where appropriate.

#### 2.2.5. Formation of Microfungal Biofilm

**Figure 1.**
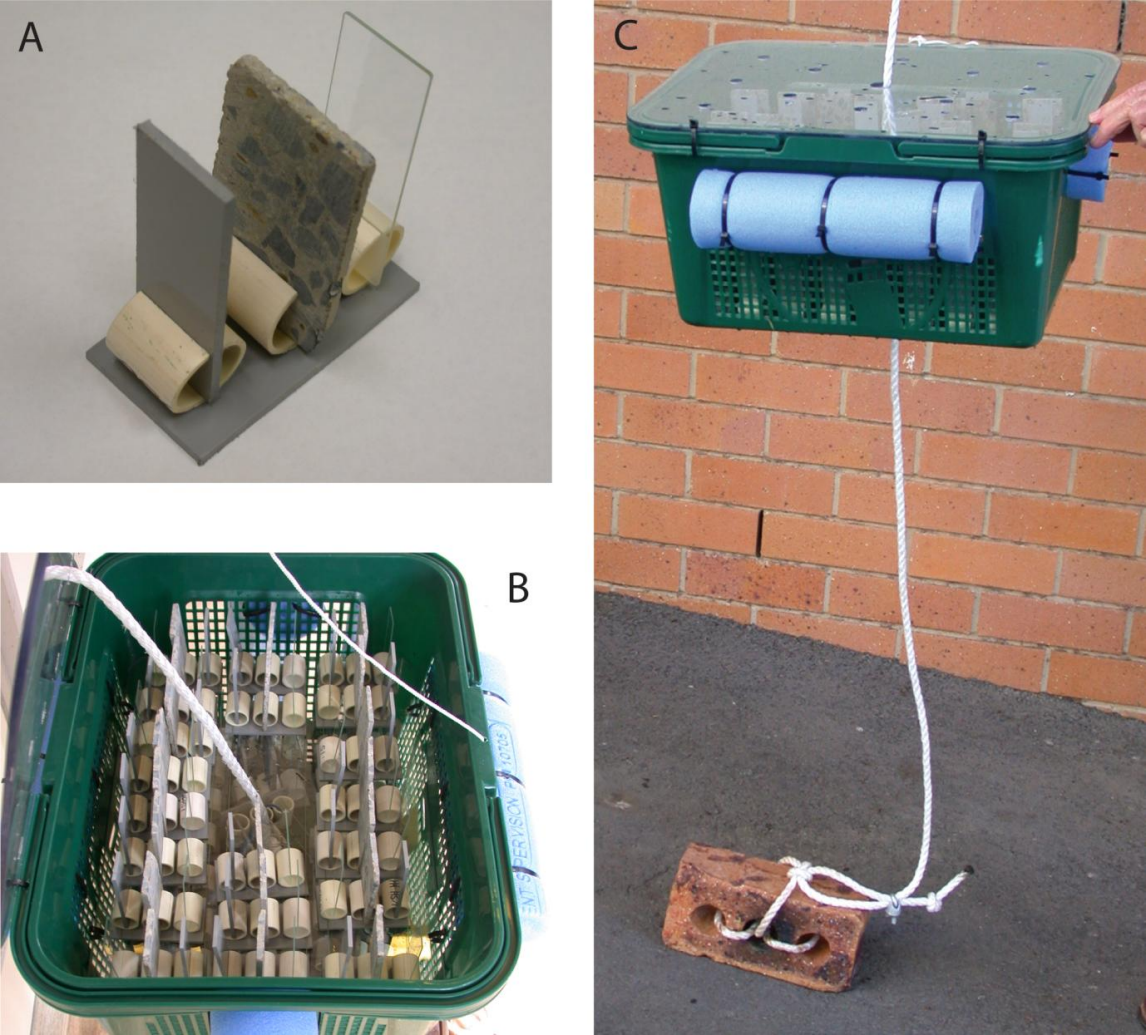
Artificial coupons and associated apparatus used to assess microfungal biofilm development. (A) Individual rack of three coupons. (B) Perforated, lidded basket holding coupon racks. (C) Completed apparatus ready for immersion in the water body of a reservoir.

The microfungal colonisation of hard surfaces within the storage and distribution system was investigated by suspending artificial coupons within the water bodies of reservoirs R3 and R4, and assessing these coupons for microfungal growth over an extended period. Coupons measuring 75 mm × 25 mm made of three materials, glass microscope slides, PVC, and concrete were held in sets of custom-made racks, each rack containing one coupon of each material (Figure 1A). The coupon racks were designed to hold each coupon vertical and apart from the other coupons to ensure a free flow of water across both surfaces of all coupons, even if a rack was dislodged onto its side. These racks were placed in lidded plastic baskets which were perforated on all sides, top and bottom (Figure 1B), and the baskets were attached to nylon ropes and suspended 1.5 m from the bottom in each of two reservoirs. The baskets of racks were held in position within the water body by means of clay house bricks, which acted as anchors securely tied to the end of the ropes. Sections of swimming pool ‘spaghetti’ were attached to the sides of the baskets with plastic zip ties to provide buoyancy (Figure 1C). The baskets were thus held at a constant level and position within the water body without fouling the internal infrastructure of the reservoir. The reservoirs are pulse-filled resulting in variable water levels throughout the day but it was ascertained that the water level was never allowed to drop below the level at which the baskets were suspended. All materials were sanitised by immersion in a 1% aqueous bleach solution (sodium hypochlorite 4% v/v active ingredient) for 20 min followed by thorough triple rinsing in clean tap water (water of the distribution system) before immersion in the reservoirs. The basket lids were secured in place with nylon cord ties prior to immersion to ensure that none of the contents could be accidentally released into the reservoir in the event of accidental upheaval of the baskets.

Three sets of two racks of coupons were removed from each of reservoirs R3 and R4 at intervals of 7 and 11 months, and from R3 at 26 months, and were transported to the laboratory in reservoir water in sterile plastic containers. The coupons, held in their racks, were gently and sequentially rinsed twice in SROW to remove residual source water. One set of coupons was used for examination under the SEM. These coupons were fixed for 48 h in a solution of 2.5% gluteraldehyde in 0.05 M KH_2_PO_4_ buffer at pH 7 [24] and subsequently air-dried in a biological safety cabinet. The PVC and concrete coupons were cut into small pieces which were then mounted on aluminium stubs, gold-sputtered, and examined under the SEM for evidence of microfungal growth and sporulation. The second set of coupons was used for microscopic examination under a light microscope. Acid fuchsin stain was applied to the glass coupons which were examined using phase contrast microscopy. A drop of Calcofluor White stain was placed on each PVC and concrete coupon, a cover slip was applied, the coupon was left to stand for 5 min and was then examined using epifluorescent microscopy. The third set of coupons was used to assess the microfungal component of the biofilm by a modification of the swabbing method described by Kelley *et al.* [17]. An area 25 mm × 25 mm on each coupon was swabbed for 30 s with a sterile cotton bud moistened in SROW. The cotton buds were then transferred to 10 mL of SROW and vortexed on a CSV90 Auto vortex mixer at setting 8 for 30 s to remove microfungal elements from the cotton buds which were then discarded. Streptomycin sulphate at a concentration of 100 mg L^−1^ was added to the biofilm solution to inhibit bacteria, and the solution was then used to make an arithmetic dilution series containing 6 mL, 3 mL, and 1 mL respectively of that solution. Each dilution series was micro-filtered and the filter membranes were placed on culture plates and incubated. Microfungal colony-forming units (CFU) were counted under a stereomicroscope and identified to genus level.

At random intervals throughout the study period, coupons were stained with DAPI and examined under epifluorescent microscopy to observe the microbial assemblages which had accumulated.

#### 2.2.6. Pipe Inner Surfaces

Sections of asbestos cement distribution pipes, *ca*. 600 mm long, were collected from five sites during maintenance operations. Care was taken to avoid contamination of the pipe interior with groundwater. The pipe sections were rinsed with clean tap water followed by SROW.

*Pipe wall fragments*—Five fragments of the inner wall were collected from the ends of each of the pipe sections. These were examined microscopically using the method described by Gonçalves *et al.* [27] for *in situ* analysis of microfungi in biofilm, but without using the oligonucleotide probe. Each fragment was mounted on a glass microscope slide with plasticine. A drop of Calcofluor White stain was applied to the surface of the fragment, a cover slip was applied, and the sample was left to stand in the dark for five min at room temperature before being examined using epifluorescent microscopy.

*Pipe wall scrapings*—Six scrape samples were taken from random positions on the inner surface of each pipe using sterile, one-sided razor blades. The scrape samples, still on the blades, were placed into individual sterile 120 mL screw-capped tubes (Sarstedt) containing 100 mL of SROW and streptomycin at a concentration of 100 mg L^−1^. Each tube was gently mixed by inversion to create an even suspension of the sample and the blade was removed with sterile tweezers. Samples 1–3 were used for enumeration of microfungal CFU. Each sample was micro-filtered in 25 mL aliquots, the filter membranes were placed on culture plates, incubated, and resulting CFU were counted and identified.

Samples 4–6 were used for visualisation of microfungal filaments, fragments, and reproductive structures. Each sample was filtered in aliquots which were of sufficient volume to ensure that each filter membrane was covered with sediment. That volume varied within a range of 50–100 mL for each aliquot and was determined by the turbidity of the sample. A piece of perspex 50 mm × 75 mm was cut for use as an enlarged microscope slide. A loaded filter membrane was placed onto this slide, several drops of Calcofluor White stain were applied to the surface, two 22 mm × 40 mm cover slips were applied side by side, and the membrane was left for 5 min in the dark. The filter membrane was then scanned under epifluorescent microscopy. The microscope stage was moved from side to side, advancing each time by one field of view until the whole area under the cover slips was examined. Adequate coverage of the filter membrane with sediment masked most if not all of the background fluorescence from the filter membrane.

#### 2.2.7. Pipe Dead Ends

Suspended sediment samples were collected from hydrants located at six pipe dead ends at scattered locations throughout the city. The hydrants were opened for 5 s to flush the hydrants and to stir up soft sediment in the dead end (set point for mains pressure is 550 kPa but this can vary up to 700 kPa). Three 1 L samples were then collected in sterile glass Schott bottles each containing 100 mg of streptomycin. At the laboratory each sample was gently mixed by inversion and split into two equal sub-samples.

The first sub-sample was filtered in 100 mL aliquots and was used for enumeration of microfungal CFU using the standard culture procedure. The second sub-sample was used for visualisation of microfungal filaments and reproductive structures. It was filtered in aliquots, the volume of which depended on the turbidity of the suspension. Each membrane was mounted and stained with Calcofluor White stain, and microfungal elements were visualised microscopically using the same procedure as described in the section “Pipe inner surfaces”

## 3. Results

### 3.1. Airborne Microfungal Spores

The numbers of microfungal spores and microfungal CFU recovered from air samples using the Burkard air samplers are in Table 1.

### 3.2. Microfungal Component of Biofilm on Coupons

*Culture plates*—The number of microfungal CFU recovered from swab samples taken from the coupons are in Table 2.

*Visualisation*—None of the glass coupons examined under light microscopy throughout the study supported recognisable microfungal growth. Likewise, epifluorescent microscopy revealed no evidence of microfungal growth on any of the PVC or concrete coupons.

Examination of the coupons using SEM showed that single-celled organisms, presumptive bacteria or possibly yeasts, were present on all materials. There was no evidence of recognisable filamentous microfungal growth on any of the coupons.

Examination of coupons stained with DAPI revealed increasingly extensive aggregations of presumptive bacteria on all coupons but no recognisable filamentous fungi were observed.

### 3.3. Pipe Inner Surfaces

Summary data are in Table 3.

**Figure 2.**
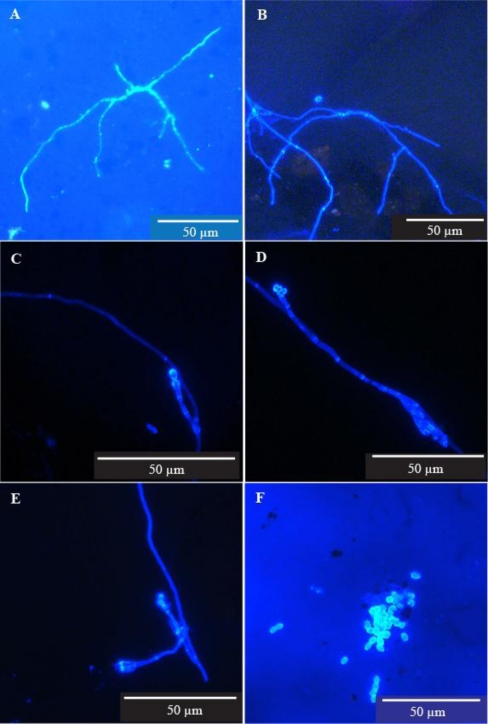
Filamentous microfungi recovered from pipe inner walls. (A) Fungal colony *in situ* on pipe wall fragment (Site P1), (B–F) Filamentous microfungi and spore aggregation recovered from pipe wall scrapings (Sites P1 and P3). The scrapings were suspended in 100 mL sterile reverse osmosis water and microfiltered (0.45 μm). The filter membranes were stained with Calcofluor White and examined with epifluorescent microscopy using a Leica DMLB microscope fitted with filter cube D (UV + violet excitation filter BP 355–425 nm wavelength and suppression filter LP 470 nm wavelength).

*Pipe wall fragments*—Microfungal growth was observed on two of the fragments, one from each of two sites (Figure 2A). Indistinct, presumptive reproductive structures were also observed on these two fragments. No data appears under Enumeration and Identification in Table 3 since the very small pipe wall fragments were visualised only. Culture was not possible.

**Figure 3.**
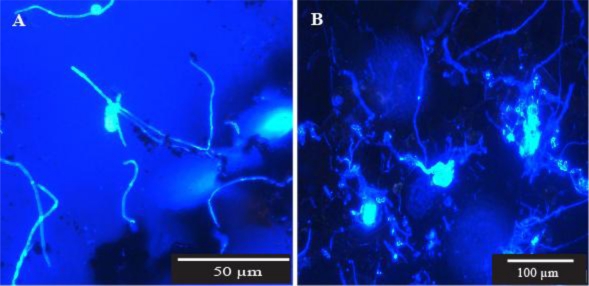
Fragmentation of filamentous microfungal colonies resulting from a disruptive method of sampling pipe biofilm. (A) Hyphal fragments (Site P1), (B) Fragmented sporulating microfungal colony (Site P4). Pipe wall scrapings were suspended in 100 mL sterile reverse osmosis water and microfiltered (0.45 μm). The filter membranes were stained with Calcofluor White and examined with epifluorescent microscopy using a Leica DMLB microscope fitted with filter cube D (UV + violet excitation filter BP 355–425 nm wavelength and suppression filter LP 470 nm wavelength).

*Pipe wall scrapings*—Some microfungi were fragmented as a result of the disruptive sampling method used. The resulting creation of numerous potential propagules is shown in Figure 3. Microfungal growth was observed in 10 of the 15 samples from the five sites. Definite reproductive structures, clearly representing several fungal genera, were observed in four of the 15 samples from three of the five sites. The hyphal elements observed were generally long, very thin and sparsely branched and reproductive structures were very sparse (Figure 2B–F).

### 3.4. Pipe Dead Ends

Summary data for each site are in Table 3.

**Figure 4.**
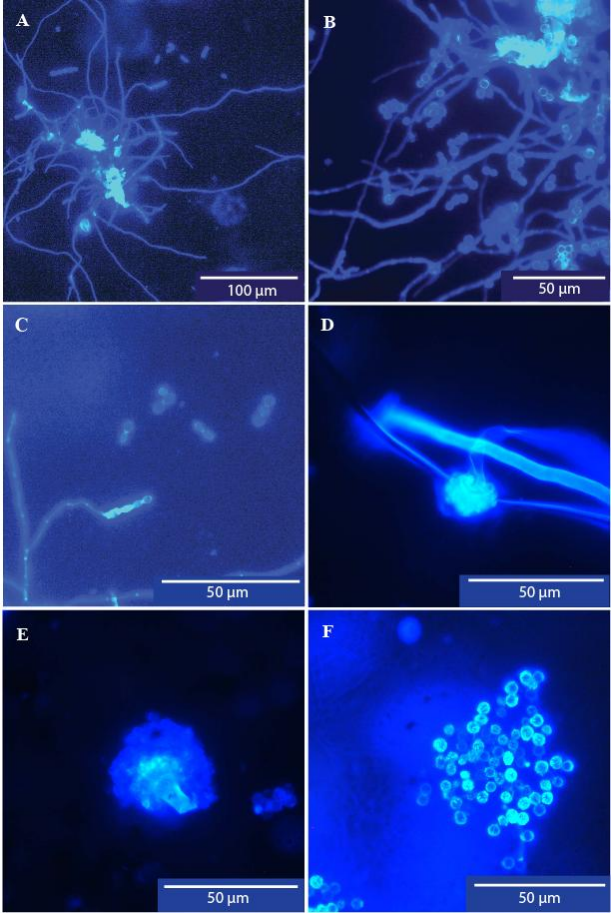
(A–F) Sporulating mycelial clumps, sporulating structures and spores of filamentous microfungi recovered from pipe dead end sediments (Sites DE1, DE5). Suspended sediment samples were collected from pipe dead ends and microfiltered (0.45 μm). The filter membranes were stained with Calcofluor White and examined with epifluorescent microscopy using a Leica DMLB microscope fitted with filter cube D (UV + violet excitation filter BP 355–425 nm wavelength and suppression filter LP 470 nm wavelength).

**Figure 5.**
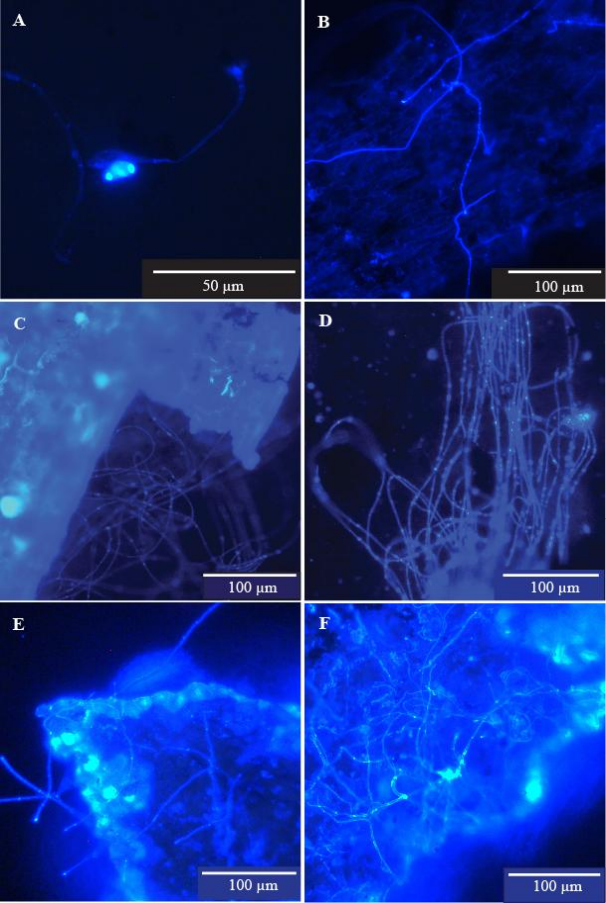
Filamentous microfungi recovered from pipe dead end sediments. (A) Spore and reproductive structure (Site DE4). (B–F) Vegetation fragments colonised by microfungi (Sites DE6, DE3 and DE6). Suspended sediment samples were collected from pipe dead ends and microfiltered (0.45 μm). The filter membranes were stained with Calcofluor White and examined with epifluorescent microscopy using a Leica DMLB microscope fitted with filter cube D (UV + violet excitation filter BP 355–425 nm wavelength and suppression filter LP 470 nm wavelength).

Microfungi were observed in all of the 18 sediment samples from the six sites. Clumps of mycelium, many containing sporulating reproductive structures, were observed as well as spore aggregations and hyphal fragments. Definite reproductive structures were observed in six of the 18 samples from four of the six sites (Figures 4 A–F, 5A). The mycelial clumps and sporulating structures were much more profuse than those seen in the pipe wall samples. A number of tiny fragments of presumptive vegetable matter colonised by microfungi were observed (Figure 5 B–F).

## 4. Discussion

The reservoirs, R3 and R4, used in this study were ideal for investigation of biofilm formation. Each is filled directly from another reservoir and those other reservoirs are automatically re-chlorinated. Neither R3 nor R4 were re-chlorinated at the time of the study and the highest numbers of microfungi recovered from the treated water distribution system during a previous study were recovered from these reservoirs [23]. Free chlorine levels in monthly samples averaged less than 0.1 ppm at both of these reservoirs throughout the period of that previous study.

### 4.1. Airborne Propagules

The only permanent avenue for airborne spores to enter the distribution system post-treatment is the storage reservoirs. All of the reservoirs in the water supply system under study are roofed, and they are all pulse-filled as demand dictates. Under normal circumstances the water level is allowed to drop during the day and the reservoir is filled at night. It is clear that the volume of water drawn out of the reservoirs by consumers would be replaced by an equivalent volume of outside air. Fungal spores contained in the air drawn into the reservoir would settle into the water body as ‘microfungal rain’, and this process would be continuous during water draw-down. The number of microfungal spores recovered on petroleum jelly-coated slides from inside air was similar to the number recovered from outside air at each of the two reservoirs. The number of microfungal colonies, and the genera, which grew on the inside air and outside air culture plates were also similar at each of the two reservoirs. The microfungal genera recovered were similar in composition to those recovered from water samples taken at the same reservoirs over an 18 month period during an earlier study [23]. The filamentous fungal genera most commonly recovered in that study were *Aspergillus*, *Cladosporium* and *Penicillium*. For that reason, and for comparative purposes, the tables presented in this study give details for those genera with the remaining filamentous microfungi shown as ‘Other filamentous fungi’. They are also expressed as a percentage of total because the different sampling methods used in the study would render use of numerical values non-comparable.

### 4.2. Formation of Microfungal Biofilm

The concrete coupons were difficult to examine under epifluorescent microscopy because of the uneven and pitted surfaces, and the autofluoresence of the material.

The recovery of small numbers of microfungal CFU by swabbing all types of coupons, together with the lack of any evidence of vegetative microfungal growth on the coupons, suggests that the former arose from allochthonous spores trapped in the water boundary layer at the solid/liquid interface of the coupons. Whether or not such spores can be categorised as biofilm microfungi is open to question. These probably originated as airborne spores, as discussed above, or as spores introduced into the reservoir by the frog *Litoria caerulea* [28]. The presence of bacterial aggregations on the coupons in the absence of fungal growth may simply represent the primary component of a very slowly developing microbial biofilm, the rate of development no doubt being controlled by the oligotrophic nature of the treated drinking water. In another part of our study, discussed in the next section, we report the discovery of fungal growth on old asbestos cement distribution pipes. When considered in conjunction with the results of the coupon experiment, this appears to support the concept of microbial succession in biofilm on drinking water pipe surfaces with increasing complexity over the duration of many years as discussed by Nagy and Olson [37]. Those authors found that as pipes aged, their inner surfaces supported a wider range of microbial groups including filamentous fungi and yeasts.

The SEM analyses of the coupons used in this study indicated that deposits on the coupons were abiotic with only scattered communities of presumptive bacteria and cyanobacteria.

### 4.3. Pipe Inner Surfaces

We provide unequivocal evidence that microfungi will grow and sporulate on the inner wall surfaces of distribution pipes in a treated water supply system (Table 3, Figure 2). It is recognised that five pipe sections is a relatively small sample size but their availability was limited to maintenance works carried out during the period when this particular study was conducted. However, the observation of microfungal filaments and hyphal fragments in 10 of the 15 scrape samples taken from those pipes, together with the reproductive structures and spores found in four of those samples, suggests that our findings are representative of the distribution system as a whole, at least as far as the asbestos cement pipe component is concerned. Further work needs to be done to ascertain whether or not microfungi are also growing on the walls of pipes made from other materials. The materials themselves, or more likely the age of the pipes, and the consequent stage of biofilm development may affect microfungal succession as discussed by Nagy and Olson [37].

While microfungal structures were observed in most of the samples they were widely dispersed, suggesting that growth on the pipe surfaces was limited. The generally long, thin, and sparsely branched filaments together with very sparse and rather simplified reproductive structures are perhaps indicative of the oligotrophic status of both the water flowing through the pipes and the very thin film of material covering the pipe surfaces.

In this part of the study, and also in the next section on pipe dead ends, we were mainly concerned with determining whether or not microfungi will grow and sporulate on the inner surfaces of water pipes and in sediment which collects in pipe dead ends. It was impossible to assess the extent of microfungal colonisation of these substrates with any degree of accuracy for the same reasons discussed by Paterson and Lima [26]. The fungi visualised in our samples were unevenly distributed and were represented by a range of structural entities including vegetative and reproductive mycelial clumps, single hyphal elements, hyphal fragments, and spore aggregations. The possible effect on the integrity of a fungal colony when the sampling method used disturbs the biofilm, such as scraping or swabbing, is illustrated in Figure 3. These photomicrographs show the fragmentation of a small sporulating colony recovered in one of our samples, and hyphal fragments recovered in another. Despite these acknowledged drawbacks we did enumerate and identify microfungal CFU from pipe surfaces and sediments using the conventional filtration and plating method for the purpose of comparison with other parts of the system. The percentage variability in genera recovery shown in Table 3 perhaps relates to the problems referred to above. Further work needs to be done to evaluate the microfungal biomass in pipe wall and sediment samples using ergosterol analysis as suggested by Paterson and Lima [26]. However, as those authors pointed out, even this method does not overcome the problem of obtaining representative samples where the fungal population is unevenly distributed as was found in our study.

### 4.4. Pipe Dead Ends

Unequivocal evidence of microfungal growth in soft sediment within water pipes, in this case dead ends, is also provided in this report (Table 3, Figures 4,5). Microfungal mycelium, often in sporulating clumps, and hyphal fragments were commonly observed in all samples. Compelling evidence that these structures originated in the sediment, rather than as particles detached from the pipe walls by the rush of water during flushing of the hydrants, was provided by the number of tiny particles of vegetation with extensive fungal colonisation which were observed (Figure 5). These particles could only have come from the disturbed sediment. The mycelial growth in the sediment samples was denser, and sporulation was more prolific, than that observed in the pipe wall samples, perhaps reflecting a higher nutritional status of the sediment.

## 5. Conclusions

This study has demonstrated that airborne spores introduced into reservoirs during drawdown periods can be an important external source of microfungal propagules found in a water supply system post-treatment.

The use of artificial coupons to assess the formation of microfungal biofilm on hard surfaces within the system has also indicated that a very slow microbial succession occurs and that microfungi are not involved in the primary colonisation.

It has been shown conclusively that a number of presumptively different genera of microfungi will grow and sporulate on the walls of asbestos cement pipes, but mycelial growth and reproductive structures are generally sparse and relatively simple.

Vegetative growth and sporulation of microfungi in the soft sediment in pipe dead ends has also been conclusively demonstrated. This suggests that any aggregation of soft sediment in the distribution system of water supply systems is a potential site for proliferation of the microfungal population. Microfungal mycelia growing on this substrate appear to be denser, and sporulation more prolific, than the growths observed in pipe wall samples.

## Figures and Tables

**Table 1.** Analysis of air samples collected inside and outside two reservoirs during March and September 2009. A Burkard Personal Volumetric Air Sampler was used to assess filamentous microfungi spore counts on Vaseline**^®^** slides, while a Burkard Personal Volumetric Portable Air Sampler was used to assess colony-forming units (CFU) of microfungi, including yeasts, on culture plates. The air volumes collected using each sampler was 150 L at each sampling event. Filamentous fungal genera recovered on culture plates are expressed as percentages of total CFU.

	**Vaseline slides**		**Culture plates**													

	In air	Out air	Inside air							Outside air						

	Total Spores No.	Total Spores No.	Total Yeast	Total filamentous fungi		*Aspergillus*	*Cladosporium*	*Penicillium*	Other fungi filamentous	Total Yeast	Total filamentous fungi		*Aspergillus*	*Cladosporium*	*Penicillium*	Other filamentous fungi
			CFU	CFU	%	%	%	%	%	CFU	CFU	%	%	%	%	%

**Reservoir R3**																
March 2009	453	511	21	328	100	13	36	32	19	28	419	100	8	24	41	27
September 2009	130	176	4	186	100	1	74	11	14	5	216	100	0	72	9	19

**Reservoir R4**																
March 2009	252	261	13	224	100	11	39	16	34	4	271	100	15	51	14	20
September 2009	236	294	11	192	100	5	68	14	13	10	190	100	1	74	6	19

**Table 2.** Microfungal colony-forming units (CFU) recovered from swabbed samples taken from 12.5 cm^2^ areas of coupon surfaces at each sampling event. Microfungal propagules were recovered by vortexing swabs in 10 mL of sterile reverse osmosis water. The suspensions were microfiltered through 0.45 μm filter membranes which were incubated on malt extract agar for 7 days at 25 °C in the dark. Filamentous fungal genera recovered are expressed as percentages of total filamentous CFU.

	Coupon material	**Reservoir R3**							**Reservoir R4**						

		Total yeast CFU	Total filamentous fungi		*Aspergillus*	*Cladosporium*	*Penicillium*	Other filamentous fungi	Total yeast CFU	Total filamentous fungi		*Aspergillus*	*Cladosporium*	*Penicillium*	Other filamentous fungi
			CFU	%	%	%	%	%		CFU	%	%	%	%	%
	
Month 7	Glass	14	14	100	21	22	7	50	44	34	100	0	15	18	67
	PVC	8	9	100	0	33	11	56	9	15	100	0	20	0	80
	Concrete	19	5	100	0	20	20	60	42	18	100	0	44	0	56
Month 11	Glass	2	30	100	30	30	7	33	0	9	100	11	56	0	33
	PVC	1	20	100	0	40	15	45	0	29	100	0	72	0	28
	Concrete	1	54	100	44	13	19	24	3	14	100	14	57	0	29
Month 26	Glass	1	21	100	5	52	5	38			Not performed				
	PVC	2	5	100	0	60	0	40							
	Concrete	1	10	100	0	30	0	70							

**Table 3.** Results of studies into the presence or absence of microfungal growth and sporulation on pipe inner surfaces and in pipe dead end sediments. Pipe fragments were mounted on glass slides with plasticine, stained with Calcofluor White and visualised under epifluorescent microscopy. Pipe scrapings were suspended in 100 mL sterile reverse osmosis water and microfiltered (0.45 μm). Fifty percent of the filter membranes were stained with Calcofluor White and examined under epifluorescent microscopy. The other 50% were cultured on malt extract agar for 7 days at 25 °C in the dark. Suspended sediment samples were collected from pipe dead ends and processed in the same way as the pipe wall scrapings. Epifluorescent microscopy was done on a Leica DMLB microscope fitted with filter cube D (UV + violet excitation filter BP 355–425 nm wavelength and suppression filter LP 470 nm wavelength).

	Site No.	1 Pipe material	Pipe Age (Yrs)	No. of samples	Visualisation only				Culture on malt extract agar					

					Number of samples in which filamentous fungi structures were observed				Total	*Aspergillus*	*Cladosporium*	*Penicillium*	Other filamentous fungi	Total yeasts
					Vegetative		Reproductive							
					(+)	(−)	(+)	(−)	%	%	%	%	%	CFU
**Pipe sections**														
*Fragments*														
	P1	A.C.	33	5	1	4	1	4	The small pipe wall fragments were visualised only. Culture was not possible.					
	P2	A.C.	35	5	1	4	1	4						
	P3	A.C.	34	5		5		5						
	P4	A.C.	25	5		5		5						
	P5	A.C.	25	5		5		5						
*Scrapings*														
	P1	A.C.	33	3	3		1	2	100	5	12	10	73	7
	P2	A.C.	35	3	1	2		3	100	9	1	18	72	17
	P3	A.C.	34	3	3		2	1	100	0	48	2	50	21
	P4	A.C.	25	3	3		1	2	100	3	20	3	74	5
	P5	A.C.	25	3		3		3	100	0	44	3	53	9
**Pipe dead-ends**														
*Sediments*														
	DE1	A.C.	26	3	3		2	1	100	39	0	6	55	0
	DE2	PVC	21	3	3			3	100	0	0	1	46	53
	DE3	PVC	20	3	3			3	100	0	0	0	97	3
	DE4	A.C.	35	3	3		1	2	100	1	20	9	43	28
	DE5	A.C.	70	3	3		1	2	100	1	6	4	87	2
	DE6	C.I.	90	3	3		2	1	100	1	37	12	49	2

1. Pipe materials; A.C. = asbestos cement; PVC = polyvinyl chloride; C.I. = cast iron.
